# Evaluations of the *in vitro* and *in vivo* antidiabetic activity of 70 % ethanolic fruit extracts of *Rosa abyssinica*

**DOI:** 10.1016/j.metop.2024.100317

**Published:** 2024-09-05

**Authors:** Mohammed Ahmed Abdul, Akeberegn Gorems Ayele, Frehiwot Teka, Worku Gemchu, Workineh Shibeshi

**Affiliations:** a Department of Pharmacology and Clinical Pharmacy, School of Pharmacy, College of Health Science, Ethiopia; b Directorate of Traditional and Modern Medicine Research, Ethiopian Public Health Institute, P.O. Box 1242, Addis Ababa, Ethiopia

**Keywords:** Diabetic mellitus, *Rosa abyssinica*, Streptozotocine, Alpha amylase, Dinitrosalisylic acid

## Abstract

**Background:**

Diabetes mellitus is becoming major health challenge with continually increasing burden. High costs of conventional medicines and numerous side effects associated with them, on the other hand, easy availability and accessibility of traditional herbal medicines calls upon experimental investigations to validate their effect on lowering blood glucose level.

**Methods:**

The dried fruit of *Rosa abyssinica* was macerated with 70 % ethanol and the extract's *in vitro* antidiabetic activity was investigated using dinitrosalisylic acid method for alpha amylase inhibitory activity. Furthermore, the *in vivo* hypoglycemic and Antihyperglycemic effects of various doses of the extract (100, 200 and 400 mg/kg) was determined on normoglycemic, glucose loaded (1500 mg/kg) and Streptozotocine (180 mg/kg)-induced diabetic mice models.

**Results:**

The acute oral toxicity study revealed the plant showed no toxic effect on swiss albino mice at 2000 mg/kg. The *in vitro* alpha amylase inhibitory activity study showed that the extract has comparable IC50 value of 21.37 ± 4.252 μg/ml with the standard drug acarbose (IC50 value of 26.72 ± 3.59 μg/ml). On the other hand, in normal mice, none of the dose levels except at 400 mg/kg significantly reduces blood glucose level. This is in contrast to the oral glucose tolerance test, which the extract produced significant reduction at 60, 90 and 120 min following glucose challenge. The 70 % ethanolic fruit extracts of *Rosa abyssinica* also experienced profound antidiabetic activity in streptozotocin-induced diabetic model. In the single-dose study, both RAFE200 and RAFE400 demonstrated a significant (P˂0.05) reduction in blood glucose levels at 1, 2, 3, and 4 h. Similarly, in the repeated-dose study, RAFE200 and RAFE400 not only significantly reduced blood glucose levels but also produced a notable improvement in animal body weight.

**Conclusion:**

The 70 % ethanolic fruit extracts of *Rosa abyssinica* have shown significant *in vitro* alpha amylase inhibition effect and an *in vivo* blood glucose level lowering effects in diabetic mice.

Therefore, this study supports the traditional use of *Rosa abyssinica* in the management of diabetes mellitus.

## 1 Background

Diabetes mellitus (DM) is one of the most disturbing and common chronic diseases of our times, causing life-threatening, disabling, and costly complications and reducing life expectancy [1]. The prevalence of non-communicable diseases continues to increase steadily, making them to prominent health concerns, with diabetes mellitus being one of the notable conditions [2]. According to the International Diabetes Federation Atlas, in 2022, there were 537 million individuals, accounting for 10.5 % of the global population, affected by diabetes. This number is projected to escalate to 784 million, or 12.5 % of the population, by the year 2045 [3]. Ethnicity, genetics, lifestyle, age and environmental conditions and/or a combination of these factors are risk factors that are major in determining a person's risk to develop T2D [4].

The autoimmune destruction of β cells, crucial for insulin deficiency leading to hyperglycemia, may arise from a combination of genetic, environmental, and immunologic influences [5]. Among the myriad of factors contributing to complications, oxidative stress plays a central role in the development of both microvascular and cardiovascular complications in diabetes [6].

Despite the limited effectiveness and tolerability faced by many oral hypoglycemic agents used in treating DM, there has been a notable rise in the utilization of natural products with antidiabetic properties. Herbal remedies are increasingly prescribed due to their efficacy, minimal side effects, and comparatively affordable nature [7]. Additionally, conventional antidiabetic medications are often very expensive and difficult to access, particularly in countries with poor healthcare systems. The intolerable side effects associated with these medications have also sparked significant interest in herbal therapies. As a result, the use of plant-based traditional remedies has become increasingly important in the management of DM [6].

**Fig. 1:**
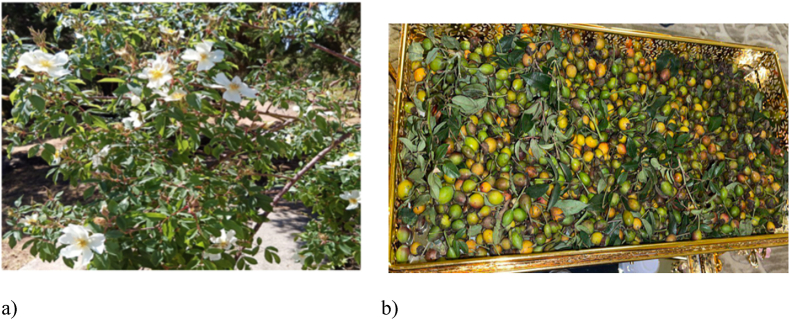
Photograph of the experimental plant, *R. abyssinica* [18]. a) The whole plant b) parts used (image was taken during plant collection).

*Rosa abyssinica (R.abyssinica)* (Figure-1*)*, a member of the Rosaceae family, holds a prominent place in Ethiopian traditional folk medicine. It is known as "Kega" in Amharic, "Qaqawwii" in Afaan Oromo, "Dayero" in Somali, and referred to as Abyssinian rose in English. It is among the herbs widely utilized in various medicinal practices across the region [8,9]. *R.abyssinica* is also prevalent in regions beyond Ethiopia and is well known in countries such as Eritrea, Sudan, Yemen, and Saudi Arabia [10]. The family, which this experimental plant belongs, includes 2500 species and 90 genera and primely found in northern temperate region. Plants in the genus “Rosa” is reported to have antineoplastic, anti-inflammatory and antioxidant, antihyperglycemic, antimicrobial and antiviral activity, as well as neuro and cardioprotective activities [11]. Likewise, *R. abyssinica* is used for management of several disease conditions including, rheumatoid arthritis, blood pressure, malaria, flu, scabies, and tuberculosis [[12], [13], [14]]. It is also reported to have antimicrobial properties and has been experimentally evaluated for its antidepressant and anti-inflammatory activities [10,12,15,16]. *R.abyssinica* also have strong antidiabetic claims that being used extensively in Ethiopian traditional medicine after macerating with traditional Ethiopian alcoholic drink called “Araki” [17]. Despite the blood glucose lowering traditional claim of this experimental plant, it is not yet experimentally evaluated against DM. Therefore, the aim of this study is to evaluate both the *in vitro* and *in vivo* antidiabetic effects of 70 *%* ethanolic Fruit Extracts of *R. abyssinica*.

## 2 Objective of the study

### 2.1 General objective

❖ To general objective of this study is to evaluate antidiabetic activity of 70 % ethanol extract of fruit of *R*. *abyssinica.*

### 2.2 Specific objective

❖ To evaluate *In Vitro* antidiabetic (α-amylase inhibitory activity) of 70 % ethanol fruit extract of *R*. *abyssinica.*
❖ To evaluate oral acute toxicity of 70 % ethanol extract of fruit of *R*. *abyssinica*
❖ To determine hypoglycemic effect of the extract in normal mice.
❖ To determine antihyperglycemic effect of the extract in glucose loaded normal mice.
❖ To determine single and repeated dose antihyperglycemic effect of the extract in streptozotocin (STZ) induced diabetic mice.
❖ To evaluate qualitative and quantify phytochemical constituents of hydroalcoholic extract of *R*. *abyssinica's* fruit.

## 3 Materials and methods

### 3.1 Drugs and chemicals

The following chemicals were used during the experiment: STZ (Sigma -Aldrich, St. Louis, MO, U.S. A), citric acid and trisodium citrate (Annexe Chem Pvt.Ltd, India), glibenclamide (Emcure Pharmaceuticals Ltd, India), glucose (TNN Group, Dalian, Liaoning, China), distilled water, chloroform (Hely Specialty Chemicals, Ankleshwar, Gujarat, India), mono and dibasic hydrogen phosphate (BDH Laboratory Supplies Ltd, England), sodium chloride, sodium hydroxide, starch, potassium sodium tartrate tetrahydrate and α-amylase (Blulux Laboratories Pvt. Ltd., Faridaban, India), bromocresol green solution and folin-Ciocalteu reagent (Ricca chemical, Arlington, TX-U.S.A), atropine, chloroform, gallic acid (Ralington pharma LLP,India), sodium carbonate, aluminum chloride, potassium acetate, quercetin(Tocris bioscience, Bristol-UK), ammonia, benzene, sulfuric acid, sodium nitroprusside, gelatin solution, lead acetate, potassium bismuth iodide(Fisher Scientific, UK), potassium mercuric iodide (May and Baker Ltd, England), 3,5-dinitrosalicylic acid (DNSA) (Sisco Research Laboratories Pvt. Ltd. Mumbai, India), acarbose (Bayer, Germany) and formalin (Genta Medical, UK).

### 3.2 Collections of the experimental plant

Ripped fruit and leaves (for identification) of *R. abyssinica* were obtained from Majete, a locality situated in north-eastern Ethiopia, approximately 328 km away from Addis Ababa. Subsequently, the plant materials were washed with tap water and left to air dry in the shade. Identification and authentication of the plant specimens were conducted by a taxonomist at the National Herbarium, College of Natural and Computational Sciences, Addis Ababa University., and specimen with voucher number: MA001, was deposited for future reference.

### 3.3 Extractions of the plant material

The washed and dried fruit of *R. abyssinica* was grossly crashed with mortar and pestle into smaller pieces. The resulted amount of powder, (746.44 g), was macerated for 3 days with 70 % ethanol (1 g–10 ml of solvent ratio) on auto shaker [19]. Thereafter, the mixture was filtered using gauze and Whatman filter paper No.1. The marc was re-macerated three times utilizing similar amount of ethanol. The combined filtrate was evaporated and concentrated using a rotary evaporator and kept in a ventilated oven at 40 °C until dried. The concentrated and dried final product of 107.09 g with a percentage yield of (14.35 %) was produced, and the extract was kept in refrigerator and whenever required fresh stock solution was prepared.

### 3.4 Experimental animals

Healthy swiss albino mice (body weight, 25–35 gm; age 7–9 weeks old) were used for the experiments. All experimental animals used for this study were obtained from the animal house of Ethiopian Public Health Institute (EPHI). Prior to beginning the experiment, the mice were placed in animal house for a period of five to seven days in order to minimize the effects of environmental stressors. The mice were then randomly assigned to different groups [20]. Mice were kept in cages made of polypropylene, in groups of six, with sawdust that was changed every 24 h. The mice were given free access to water both before and throughout the experiment. The handling and euthanasia of animals were conducted in accordance with internationally recognized guidelines. Additionally, the Institutional Review Board (IRB) of the School of Pharmacy approved all procedures.

### 3.5 Preliminary phytochemical screening

A preliminary qualitative phytochemical study was carried out on 70 % ethanol *R. abyssinica's* extract using standard phytochemical reagents for the presence or absence of secondary metabolites including alkaloids, steroidal molecules, phenolic compounds, flavonoids, saponins, and tannins [21,22].

### 3.6 Test for alkaloids

Two grams of the crude extract was placed in a test tube and mixed with 10 mL of 1 % HCl for 30 min in a water bath and then filtered. The filtrate was divided into two separate test tubes. Into each tube, 1 mL of Dragendorff's reagent (potassium bismuth iodide solution) and 1 mL of Mayer's reagent (potassium mercuric iodide solution) were respectively introduced and thoroughly mixed by shaking. Yellowish orange precipitate (for Dragendroff's reagent) and whitish/cream precipitate (for Mayer's reagent) indicated the presence of alkaloids.

### 3.7 Test for phenols

Test for phenols was conducted using the ferric chloride test. Accordingly, 1 mL solution of an extract was taken and placed into a test tube. Then, 1 % gelatin solution containing sodium chloride was added and shaken. Formation of bluish-black color indicates the presence of phenols.

### 3.8 Test for flavonoids

Employing the lead acetate test to detect flavonoids, 1 mL of the extract was transferred into a test tube. Subsequently, five drops of lead acetate were added to the tube and thoroughly mixed by shaking. The appearance of a yellow precipitate indicated the endpoint, confirming the presence of flavonoids.

### 3.9 Test for tannins

Screening for this particular secondary metabolite was conducted using gelatin test. Accordingly, 1 mL of extract was taken and placed in a test tube. Following this, 1 % gelatin solution containing sodium chloride was added and shaken. In this test, appearance of white precipitate indicates the presence of tannins.

### 3.10 Test for saponins

Test for saponins was performed using the froth formation**.** Accordingly, 0.5 g of 70 % *R. abyssinica* crude extract was dissolved in 10 ml of distilled water in a test tube. The test tube was then shaken vigorously for 30 s the formation of “honey comb” froth that persisted indicated the presence of saponins.

### 3.11 Test for glycosides

For presence or absence of glycosides, 1 ml of an extract was taken, and then an equal volume of sodium nitroprusside was added followed by few 3 ml of sodium hydroxide solution and shaken. Finally, formation of pink-to-blood-red precipitate signifies the existence of cardiac glycoside.

### 3.12 Test for steroids and triterpenoids

In this particular test, 100 mg of 70 % ethanol extract of *R.abyssinica* was dissolved in 2 ml of chloroform, shaken, and filtered. Few drops of concentrated sulfuric acid were added to filtrate, shaken, and allowed to stand. Development of golden-yellow precipitate indicated the presence of triterpenes.

### 3.13 Test free anthraquinones

100 mg of the 70 % ethanol extract of *R. abyssinica* was shaken vigorously with 10 ml of benzene and the extract was filtered. The filtrate was treated with 5 ml of 10 % ammonia solution and was shaken. The formation of pink/red color in the ammonia phase was considered as positive free anthraquinones.

## 4 Quantitative analysis for selected secondary metabolites

### 4.1 Analysis of total alkaloid content

Total Alkaloid Content (TAC) of 70 % ethanol extract of *R. abyssinica* was calculated using bromocresol green (BCG), or bromocresol green solution, atropine standard solution and phosphate buffer with slight modification [[23], [24], [25]]. Accordingly. A 1000 ml solution of bromocresol green solution was made by boiling 70 mg of bromocresol green, 3 ml of 2N NaOH, and 5 mL of distilled water until the BCG was completely dissolved. Disodium phosphate (4.543 gm Na_2_HPO_4_) and sodium dihydrogen phosphate (0.192 gm NaH_2_PO_4_) were combined with 1 L of distilled water to create phosphate buffer solution (pH 4.7). A solution containing different concentrations of pure atropine ranging from 0.4 ml (0.04 mg/ml) to 1.2 ml (0.12 mg/ml) was prepared all dissolved in water. To each concentration, 5 mL of phosphate buffer and BCG solution were added, followed by vigorous shaking. The volume of each standard atropine concentration was then adjusted after rinsing with 1, 2, 3, and 4 ml of chloroform. Finally, the solutions were collected into 10-ml volumetric flasks.

Using UV spectrophotometry, the absorbance was measured at 470 nm in comparison to the blank that was made as previously but without atropine. To separate alkaloids, 5 g of *R. abyssinica* sample extract were dissolved in 2 N hydrochloric acid (HC) and subsequently filtered using whatman filter paper. After that, 1 mL of the filtered solution was put into a separatory funnel and three times rinsed with 10 mL of chloroform, which was discarded, and pH of the solution in the separatory funnel was titrated to neutral pH with 0.1 N NaOH. After that, the solution was mixed with 5 mL of BCG solution and 5 mL of phosphate buffer, and it was shaken. Vibrant shaking was used to extract the residual complex using 1, 2,3 and 4 mL of chloroform. The extracts were collected in a 10-ml volumetric flask and diluted it. At 470 nm, the complex's absorbance in chloroform was determined. The assay was performed in triplicate manners, with the mean value being determined.in milligrams of atropine per (mgATE/g) gram of extract.

### 4.2 Analysis of total phenolic content

Total phenolic contents of 70 % ethanol extract of *R. abyssinica* was determined using a Folin-Ciocalteu (FC) colorimetric method [26]. Accordingly, different concentrations of a standard phenol gallic acid solution (5, 10, 20,25,30,40 and 50 μg/mL) were prepared initially. Thereafter, 2.5 mL of purified water was poured into 1 mL of diluted extract (5 gm in 20 ml DW), mixed with 0.5 ml of FC reagent, and incubated for 10 min before adding 2 mL of 7.5 % sodium carbonate and then kept it in the dark for 30 min. Finally, the resulted bluish colored solution's absorbance was determined at 765 nm. Similar steps were used for each gallic acid concentration: 2.5 ml of DW,0.5 ml of FC, 2 mL of sodium carbonate was and after the proper incubated time was over, the resulted blue/greenish solution absorbance at 765 nm was measured and the calibration curve was made. All measurements were made the outcome was given as milligrams of gallic acid equivalents (mgGAE/g) per gram of extract.

### 4.3 Analysis of total flavonoid content

Total Flavonoid content (TFC) of 70 % ethanol extract of *R. abyssinica* was determined by aluminum chloride colorimetric method as described elsewhere [27]. Accordingly, different concentrations of quercetin solution in methanol were prepared (15.62, 100, 200, 300, 400 and 500 μg/mL). Thereafter, 0.5 mL of sample from each concentration of the standard solution were mixed in a separate test tube with 0.1 mL of 10 % aluminum chloride, 0.1 mL of 1M potassium acetate, and 2.8 mL of distilled water. After 30 min of the incubation period, the absorbance of the solution was measured at 415 nm against the blank prepared as above without quercetin and the standard curve with linearity was prepared. Instead of using 1 mL of gallic acid, test tube with 1 mL of plant extract solution with a concentration of 1 mg/ml was prepared and mixed with the same reagents as described previously. Following this, absorbance was measured at the same wavelength as before. For each investigations, measurement was done three times and the mean value was recorded. Finally, the result was expressed as milligrams of quercetin equivalent per gram of extract (mgQE/g).

## 5 *In vitro* antidiabetic effect study

### 5.1 Determinations of α-amylase inhibition activity

The 3.5- dinitrosalicylic acid (DNSA) technique was used to conduct the α-amylase inhibition activity [28]. Fruit extract of *R. Abyssinica* and positive standard drug, acarbose, were dissolved in a buffer ((Na_2_HPO_4_/NaH_2_PO_4_ (0.02 M), NaCl (0.006 M) at pH 6.9) to prepare a concentration ranging from 100 to 1000 μg/ml of extract and acarbose. Accordingly, a volume of 200 μl of α-amylase solution, 2 units/ml, was mixed with 200 μl of each concentration of the extract and acarbose and was incubated for 10 min at room temperature. After that, in each tube 200 μl of the 1 % starch in water (w/v) solution was added, which then incubated for 3 min. Subsequently, 200 μl of DNSA reagent (12 g of sodium potassium tartarate tetrahydrate in 8 ml of 2M NaOH and 20 ml of 96 mM of 3,5-dinitrosalicylic acid solution) was added to terminate the reaction, and it was then boiled for 10 min at 87 °C in oven. Following this, the mixturewas allowed to cool to room temperature before being diluted with 5 ml of distilled water. Finally, the absorbance was measured at 540 nm using a UV–Visible spectrophotometer.

The control with 100 % enzyme activity was made by substituting the plant extract/acarbose solution with 200 μl of buffer. Likewise, buffers devoid of enzyme solution, and various concentrations of inhibitors (extract or acarbose) without enzyme were prepared following the aforementioned procedure. The α-amylase inhibitory activity is expressed as percent inhibition and was determined using the equation given below (Formula-1). Finally, the % α-amylase inhibition was plotted against the extract and acarbose concentration and linearity was obtained. Half-maximal inhibitory concentration (IC50) value, which is the concentration of sample required to inhibit 50 % α-amylase activity, was obtained from the graph for both extract and standard drug. Measurement was carried out three times for both, acarbose and extract.

(1) Inhibition (%) = ((*Ac*−*Acb*) −(*As*−*Asb*))/((*Ac*−*Acb*)) * 100

where Ac refers to the absorbance of positive control (enzyme and buffer); Acb refers to the absorbance of control blank (buffer without enzyme); As refers to the absorbance of sample (enzyme and inhibitor); and Asb is the absorbance of sample blank (inhibitor without enzyme).

### 5.2 Acute oral toxicity study

The acute toxicity of the *R. abyssinica* was assessed in accordance with OECD Guideline 425 [29]. For this investigation, five female mice aged between 6 and 8 weeks, with an average weight of 32 g, were employed. Prior to and following the administration of the extract, all mice underwent a fasting period of 4 h and 2 h, respectively.

Regarding the plant extract, it was initially allowed to be dissolved in water with the help of magnetic stirrer. Since no fatalities were observed in the first mouse within 24 h, the same dose of the extract was administered to the remaining 4 mice. The animals were observed continuously for 4 h with 30 min interval during the first 24 h. Observation was conducted to assess general signs and symptoms of toxicity, including abnormalities in skin, tremors, changes in fur color, convulsions, salivation, diarrhea, coma, and mortality. This observation period lasted for a total of 14 days.

### 5.3 Blood glucose level measurement

In all animal models, blood samples were taken aseptically by cutting off the tip of the tail in order to measure the blood glucose level (BGL). BGL tests were performed using glucometer standard strip/kits (Smart lab,Gmbh, Germany). Measurements were taken three times, and the average result was recorded. During each measurement, 70 % ethanol was applied with cotton to prevent infection at the tip of the tail [17].

### 5.4 Induction of experimental DM

In this experiment, diabetes was induced using a single high dose of STZ. The dose required to produce DM in rodents depends on, type of animal and species utilized, and the company where this chemical is made [[30], [31], [32]]. Therefore, a preliminary investigation was conducted across three dosage levels (150 mg/kg, 180 mg/kg, and 200 mg/kg) to determine at what level the product would be able to induce DM. Subsequently, the dosage of 180 mg/kg was chosen due to its favorable outcomes. For the main experiment, eighty male mice were acclimated for five days before the experiment began. On the first day, 4 h before STZ administration, food was removed across all animal but water was provided as usual. First, STZ was dissolved in freshly prepared 0.1M citrate buffer, which had its pH adjusted to 4.5. Thereafter, based on the dosage, STZ was administered to mice via intraperitoneal injection [33].

The mice were given standard food pellets and 10 % sucrose water and they were attentively observed for 12 h for signs of hypoglycemia. On the third day of the experiment, the 10 % sucrose changed and normal water was given instead. Mice were allowed to fasted for about 6 h. The BGL was then measured using a tail-vein blood sampling technique via glucometer and mice were considered as diabetics if their FBG level was 200 mg/dl or higher [33,34].

## 6 Grouping and dosing

### 6.1 Model, dose and groups

In both normoglycemic and oral glucose tolerance test (OGTT) assessments, animals of both sexes were randomly assigned to five groups: negative control, positive control, and three test groups, each consisting of 6 animals per group. Group I served as the negative control (NC), while Group II served as the positive control (GL5). Similarly, once DM inductions were confirmed in STZ models, the laboratory animals were divided into six groups. The same grouping was made except in this case one additional group, normal control (NOC) receiving 10 mg/kg of distilled water (DW) was added. In all models the test group received 100 mg/kg of 70 % % ethanoic fruit extracts of *R. abyssinica* (RAFE100), 200 mg/kg (RAFE200) and 400 mg/kg (RAFE400) while negative control and the positive control group received DW(10 mg/kg) and 5 mg/kg of glibenclamide (GL5) respectively.

### 6.2 Assessment of hypoglycemic activity in normal mice

Assessment of hypoglycemic activity in normal mice was done as described elsewhere [35]. In this model, each group of animals were allowed to fast for 14 h with free access to water. Subsequently, groups received either DW, glibenclamide, or varying doses of the extract according to the experimental design. BGL were then measured at 0, 1, 2, 3 and 4 h after treatment.

### 6.3 Oral glucose tolerance test

The OGTT was conducted employing mice that had fasted overnight (14 h). Following the fasting period, animals were randomly allocated into five groups, with six mice per group. Baseline BGL were measured immediately before administering each agent based on their respective groupings. Subsequently, DW, the extract, and a standard drug were given. Thirty minutes post administrations of each agent, the animals were orally administered a glucose solution at a dosage of 2.5 g/kg. Blood glucose levels were then measured at 30, 60, and 120 min following glucose solution [36].

**Evaluations of the anihyperglycemic effect of 70 % ethanolic fruit extracts of*****Rosa abyssinica*****in STZ-induced diabetic mice**.

### 6.4 Single-dose study

Single-dose antihyperglycemic effect of 70 % fruit extract of *R. abyssinica* extract was carried out 5 days after STZ injection on STZ-induced diabetic mice. Following a 14-h overnight fast, blood sample was taken at 0, 1, 2, 3 and 4 h-just before giving DW, standard drug and three doses of extract (100 mg/kg,200 mg/kg and 400 mg/kg) of *R. abyssinica* as per grouping [37].

### 6.5 Repeated doses study

In repeated dose study, based on their grouping, diabetic mice were given DW, standard drug or extract for 3 weeks. The non-diabetic group or the normal control (NOC) was also administered DW for the same time. The blood glucose lowering effects of extract was then determined by measuring FBG every seven days for three weeks. BGL of diabetic mice were measured just before starting treatment on the 1st day of treatment (3 days after STZ injection) as baseline, and then on the 1st, 2nd and 3rd week following fasting for 14 h [38].

### 6.6 Determination of body weight

The effects of STZ on body weight reduction and the enhancement of body weight change by extract and standard were evaluated. Before STZ administration, the mice in the control group and all treated groups had their body weight measured as baseline and body weight change was measured in the 1st, 2nd and 3rd week of repeated dose study [20].

### 6.7 Data analysis

All statistical analyses were performed using international business machine of statistical package for the social Sciences, (IBM SPSS), version 26 for windows (SPSS inc, Chicago, Illinois, USA). For *in vitro* studies, independent sample *t*-test was employed. It was used to assess whether there was a statistically significant difference in α-amylase inhibition activity between the extract and the standard drug, Acarbose. For *in vivo* antidiabetic effect, statistical differences between groups was analyzed by one-way analysis of variance (ANOVA) followed by Tukey post hoc test. In all cases results were expressed as mean ± standard error mean (SEM). P-values less than 0.05 were considered as statistically significant.

## 7 Results

### 7.1 Preliminary phytochemical screening

The preliminary phytochemical screening of 70 % ethanol fruit extract of *Rosa abyssinica.* Showed the presence of all tested secondary metabolites (Table-1).

**Table 1:** 70 % ethanol fruit extract of *R. abyssinicaיs* secondary metabolites.

Screened secondary Metabolites	Test reagents/methods	Absence/presence
Alkaloids	Dragendroff's/Mayer's test	presence
Phenols	Ferric chloride test	presence
Flavonoids	Lead acetate test	presence
Tannins	Gelatin's test	presence
Saponins	Honey comb test	presence
Glycosides	Legals test	presence
Steroids/triterpenoids	Salkowsk's test	presence
Anthraquinones	anthraquinones	presence

**Table-2:** IC50 values of the standard drug and plant extract in triplicate measurements.

Agent	IC50			Mean of Ic50	P-value	95 % CI
	Round I	Round II	Round III			
Acarbose	27.81	20.03	32.32	26.72 ± 3.59	0.392	[-10.27, 20.96]
RAFE	25.73	12.87	25.52	21.37 ± 4.252		

**Note:** Data are expressed as mean ± S.E.M; Analysis was performed by independent sample *t*-test; RAFE, *Rosa abyssinica* fruit extract; CI, confidence interval.

**Table 3:** Effect of 70 % ethanol fruit extract of *R*. *abyssinica* on oral glucose tolerance test.

Group	Blood glucose concentration in (mg/dl)					% BGL increment
	0 Minute	30minuts	60minuts	90 min	120 min	At 30 min
NC	110.17 ± 1.66	228.50 ± 7.97	175.17 ± 2.18	163.83 ± 1.327	126.67 ± 2.18	107.4
GLI5	106.50 ± 0.88	173.67 ± 5.20a**	113.17 ± 3.31a***	97.83 ± 4.04a***	72.83 ± 1.95a***	39.59
RAFE100	106.17 ± 0.83	213.00 ± 16.77	170.83 ± 1.27	155.50 ± 5.494	121.83 ± 1.85	84.92
RAFE200	104.50 ± 2.15	194.83 ± 0.47	160.83 ± 2.18a**	143.17 ± 1.30a**	114.33 ± 2.86a**	84.52
RAFE400	107.83 ± 2.85	186.17 ± 1.86a*	160.33 ± 1.20a**	112.00 ± 3.44a***	105.83 ± 2.38a***	80.68

Note: Data are expressed as mean ± S.E.M; n = 6 for each treatment; Analysis was performed by one way ANOVA. a, compared to negative control; NC, negative control treated with distilled water; GLI, glibenclamide; RAFE, Rosa abyssinica fruit extract; number followed by RAFE and GLI indicates dose/ in mg/kg; h, hour; *p < 0.05; **p<0.01 ***;p < 0.001.

## 8 Quantitative analysis for selected secondary metabolites

### 8.1 Analysis of Total Alkaloid Content

**Figure-2:**
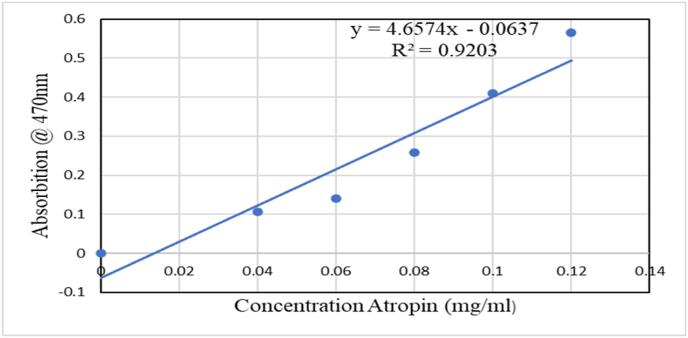
Atropine standard absorbance graph.

The standard absorbance graph for atropine was expressed in a linear line with equation Y = 4.657X + 0.0637 and a correlation coefficient (R^2^) of 0.9203 (Figure-2). The average triplicate absorbance for the sample resulted in 0.452 and total Alkaloid content (TAC) 83.37 mg ATP/g dry extracts.

### 8.2 Determinations of total phenolic content

**Figure-3:**
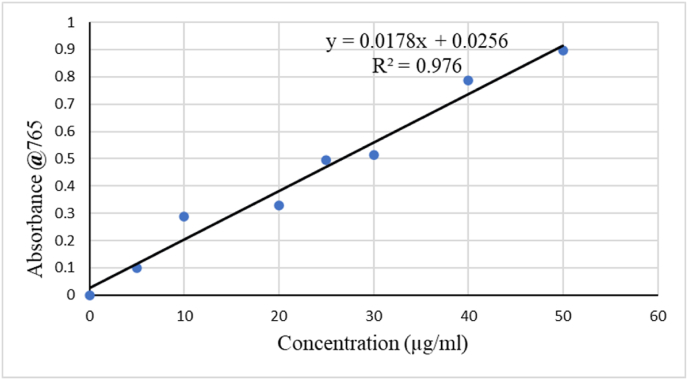
Gallic acid standard absorbance graph.

The standard absorbance graph for gallic acid was expressed with equation Y = 0.0178X + 0.0256 and a correlation coefficient (R^2^) of 0.976 (Figure-3). Furthermore, the average triplicate absorbance for the sample resulted in 0.82 and total phenolic content (TPC) of 892 mg GAE/g dry extract.

### 8.3 Determinations of flavonoid content

**Fig. 4:**
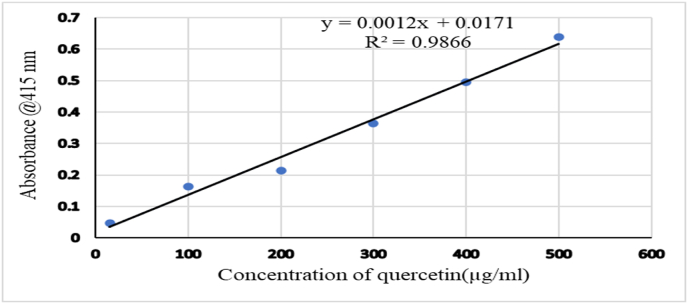
Quercetin standard absorbance graph.

The standard absorbance graph for Quercetin was expressed in a linear line with equation Y = 0.0012X + 0.0171 and a correlation coefficient (R2) of 0.9866 (Figure-4). The absorbance for the sample resulted in 0.361 and Total Flavonoid Content (TAC) of 286.58 mg QC/g dry extract.

### 8.4 *In vitro* α-amylase inhibitory activity

**Fig. 5:**
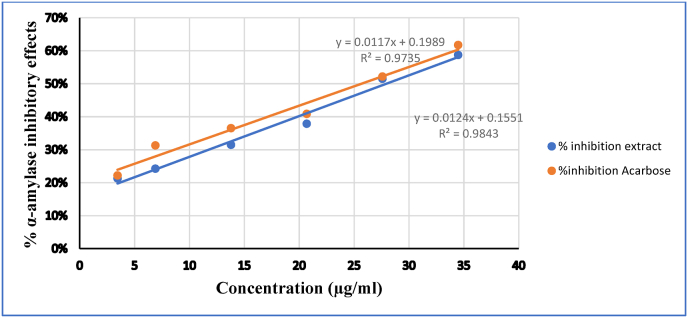
⁚ Concentration vs percentage α-amylase inhibitory effects of the 70 % ethanol fruit extract of *R. abyssinica* and Acarbose The average IC50 values from triplicate measurements were 26.72 ± 3.59 μg/ml and 21.37 ± 4.252 for the standard drug and plat extract respectively indicating no significant difference between the experimental plant and the standard drug (Table-2).

Figure-5 shows how IC50 can be derived just by taking the values of the measurement of α-amylase inhibition activity the first round in triplicate measurement and the calculated IC50 is 25.76 g/ml for the standard drug and 27.81 μg/ml for the experimental plant.

### 8.5 Acute toxicity study

In the acute toxicity study, the administration of a single dose of 2000 mg/kg of the 70 % ethanol fruit extract of *R. abyssinica* exhibited no toxic effects on Swiss albino mice. After 24 h, the animals showed tolerance and displayed no observable signs or symptoms of toxicity, including abnormal changes in skin and fur color, tremors, convulsions, salivation, diarrhea, or coma. Furthermore, there were no instances of mortality observed at the end of the two-week observation period.

Based on the acute toxicity study, which shows that the extract is safe at a dose of ≥2000 mg/kg, the doses for the main study are typically chosen as 1/20th, 1/10th, and 1/5th of this dose, resulting in doses of 100, 200, and 400 mg/kg, respectively. This approach has been commonly used in previous studies when the extract is considered safe at these levels [20,39].

### 8.6 Hypoglycemic effects of 70 % ethanol extract in normal mice

**Fig. 6:**
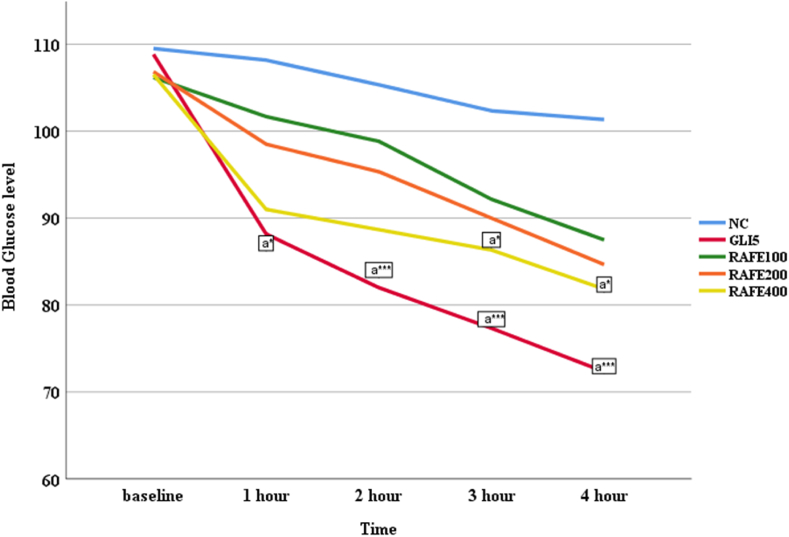
Effect of 70 % fruit extract of *R. abyssinica* on fasting BGL of normoglycemic mice **Note:** Data are expressed as mean ± S.E.M; n = 6 for each treatment; Analysis was performed by one way ANOVA; a, compared to negative control; NC, negative control treated with distilled water; GLI, glibenclamide; RAFE, *Rosa abyssinica* fruit extract; number followed by RAFE and GLI indicates dose/in mg/kg; h, hour; *p < 0.05; **p < 0.01 ***; p < 0.001.

The baseline fasting BGL (0h) of all groups showed no apparent difference between them (Figure-6). Except the RAFE400 that showed significant difference in reduction of BGL at 3 and 4 h (P˂0.05) compared with the negative control, none of the extracts dose produce any significant deference compared with the negative control group. On the other hand, a significant BGL reduction was observed in GLI5 group at all-time points.

### 8.7 Antihyperglycemic effect of extract in oral glucose tolerance test

Glucose loading resulted in elevated BGL levels across all groups, with no significant differences between them (Table-3). Thirty minutes post administration, a maximum increase achieved with the NC (107.40 %) but the standard treatment group (p < 0.01) and mice treated with the highest dose of the extract, RAFE400 (p < 0.05), exhibited a significant decrease in BGL, which persisted for up to 120 min. Additionally, animals treated with the middle dose of extract experienced a reduction in BGL at all time points starting at 60 min since glucose loading.

### 8.8 Induction of experimental type 2 diabetes mellitus in mice

Out of 80 male mice administered STZ (180 mg/kg) to induce diabetes, 46 mice (with an induction rate of 57.5 %) developed blood glucose levels over 200 mg/dl within three days. Diabetic animals were randomly selected and grouped into five groups (6 mice each) and single and repeated dose studies were performed.

## 9 Effects of administration of extracts to streptozotocin -induced diabetic mice

### 9.1 Single dose antihyperglycemic effect

The STZ-induced diabetic mice exhibited a significant increase in FBG as compared to normal control groups before the initial treatment of different doses of extract or the standard drug (Table-4). Following the commencement of the treatment, although the lowest dose of extract failed to reduce FBG, RAFE200 and RAFE400 showed a significant reduction in BGL at the1^st^ and 2nd hr (p < 0.05) and these effect continues to be apparent at the 3rd and 4th h (p < 0.001). Likewise, the standard drug witnessed a significant FBG lowering effect at all-time points with maximum effect recorded at the 4th hr. Moreover, while the standard drug took 2 h to reduce FBG level back to normal, RAFE200 and RAFE400 started to have the same effect at the 3rd hr.

**Table 4:** Antihyperglycemic activity of Single dose 70 % ethanol fruit extract of *R*. *abyssinica* in STZ-induced diabetic mice.

Group	Blood glucose level concentration in (mg/dl)				
	Baseline	1hr	2hrs	3hrs	4hrs
NOC	99.50 ± 2.778	97.33 ± 1.838	94.17 ± 2.50	91.33 ± 2.216	89.50 ± 2.895
NC	364.83 ± 48.11a***	359.5 ± 44.62b***	348.00 ± 40.899b**	335.33 ± 41.175b***	321.83 ± 43.41b***
GLI5	380.50 ± 12.92a***	203.17 ± 8.71a*b*	111.17 ± 3.29b**	48.50 ± 8.011b***	41.33 ± 7.61b***
RAFE100	413.00 ± 24.54a***	335.33 ± 7.56	301.17 ± 3.13	274.50 ± 7.54	243.67 ± 10.80
RAFE200	382.17 ± 36.98a***	276.83 ± 4.41a**b*	176.67 ± 5.59a*b**	130.33 ± 2.41b***	106.17 ± 2.57b***
RAFE400	383.33 ± 10.2a***	245.33 ± 7.71a**b*	139.67 ± 3.31a*b**	100.50 ± 2.60b***	89.17 ± 2.70b***

Note: Data are expressed as mean ± S.E.M; n = 6 for each treatment; Analysis was performed by one way ANOVA. a, compared to normal control group; b, compared to negative control; NC, negative control treated with distilled water; GLI, glibenclamide; RAFE, Rosa abyssinica fruit extract; number followed by RAFE and GLI indicates dose/ in mg/kg; h, hour; *p < 0.05; **p<0.01 ***;p < 0.001.

### 9.2 Repeated dose antihyperglycemic effect

Weekly FBG level was measured to determine the repeated dose effect of *R.abyssinica* in STZ-induced diabetic mice (Figure-7). Multiple comparison revealed that RAFE200 and RAFE400 showed a significant FBG reduction at 1st (p < 0.05), 3rd and 4th weeks (p < 0.001). On the other hand, the lowest does of extract experienced noticeable FBG reduction (p < 0.05) only at 3rd week. Similarly, mice treated with the standard drug (GLI5) witnessed a significant FBG reduction at all-time points (p < 0.001). While the negative control group exhibited diabetic status consistently across all time points, GLI5 and RAFE400 by the 2nd and 3rd weeks and RAFE200 at the end of the experiment demonstrated a tendency to normalize FBG levels. Conversely, mice treated with lower doses of the extract consistently displayed significantly higher FBG levels compared to the normal control group at all-time points (p < 0.05).

**Figure- 7:**
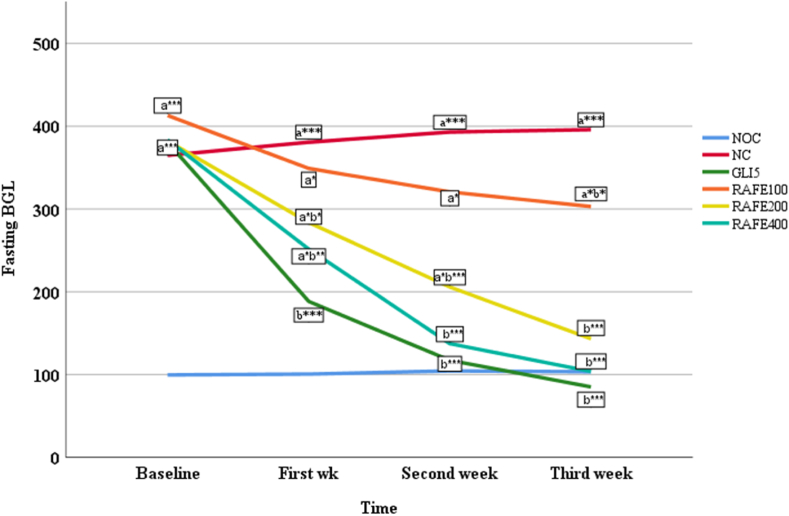
Antihyperglycemic activity of repeated doses of 70 % ethanol fruit extract *of R. abyssinica* in STZ-induced diabetic mice **Note**: Data represents mean ± S.E.M; n = 6 for each treatment; Analysis was performed by one way ANOVA; a, compared to normal control; b, compared to negative control; number followed by RAFE and GLI indicates dose in mg/kg; NOC, normal control treated with distilled water; NC, negative control treated with distilled water; GLI, glibenclamide; RAFE, *Rosa abyssinica* fruit extract; number followed by RAFE and GLI indicates dose/in mg/kg * <0.05; ** <0.01; *** <0.001.

### 9.3 Effect of 70 % ethanol extract of *Rosa abyssinica* fruit on body weight change

There was no significant body weight difference among all six groups initially (t = 0) (Table-5). In the weeks followed, however, the NC showed significant body weight reduction compared to normal control groups at the 1st (p < 0.01), 2nd and 3rd week (p < 0.01). Similar patterns except in first week in animals receiving RAFE100 was observed. RAFE200 and RAFE400, showed significantly less body weight reduction at the 2nd (p < 0.01) and 3rd week (p˂0.001) of treatment periods compared to negative control groups. Attenuations of body weight decline was also observed in all but at 1st week in groups which were on the standard drug (GLI5).

**Table-5:** Effect of 70 % ethanol extract of *R. abyssinica* fruit on mice body weight.

Group	Body weight in gram			
	Baseline	Week 1	Week 2	Week 3
NOC	31.1333 ± 0.70	31.800 ± 0.55	32.300 ± 0.56	32.950 ± 0.55
NC	30.1333 ± 1.48	24.533 ± 0.38a**	21.617 ± 0.56a***	18.467 ± 0.24a***
GLI5	28.1833 ± 1.08	27.083 ± 1.01	28.773 ± 0.872b***	28.183 ± 1.11b***
RAFE100	31.5833 ± 1.98	27.483 ± 1.67	24.350 ± 1.37a***	21.43 ± 0.4551a***
RAFE200	29.7500 ± 1.38	26.750 ± 1.35	27.500 ± 1.21b**	28.350 ± 1.18b***
RAFE400	31.5500 ± 1.52	30.467 ± 1.53	29.635 ± 1.55b**	30.717 ± 1.33b***

**Note**: Data represents mean ± S.E.M; n = 6 for each treatment; Analysis was performed by one way ANOVA. a, compared to normal control; b, compared to negative control; NOC, normal control treated with distilled water; NC, negative control treated with distilled water; GLI, glibenclamide; RAFE, Rosa abyssinica fruit extract; number followed by RAFE and GLI indicates dose in mg/kg *< 0.05;** < 0.01 ;*** < 0.001.

## 10 Discussion

This study was conducted to evaluate the antidiabetic activity of 70 % ethanolic extract of *R. abyssinica* both vitro and *in vivo*. The experimental plant, *R.abyssinica*, is the only *rose* native to Africa which belongs to the big Rosaceae family [16]. In this family of plants, the genus of *Rosa*, are recognized to have cardioprotective, neuroprotective antihyperglycemic, anti-inflammatory and laxative properties [40]. *R. abyssinica* locally known as “kega” is an evergreen shrub that holds a high value in the Ethiopian folk medicine. It is used for the treatment of, tuberculosis, hypertension, rheumatic pain, scabies, cough, and DM [41]. Considering how traditionally peoples use the plant, macerating with a local alcoholic drink called “Araki”, for medicinal purpose specially diabetes and in line with the traditional claim, the solvent 70 % of ethanol was used for extraction [42].

In this study, before commencing an *in vivo* trial, the antidiabetic effect of 70 % ethanolic fruit extracts of *R.abyssinica*.was examined *in vitro* by measuring the α-amylase inhibitory activity. Inhibition of starch-metabolizing enzymes such as α-amylase is one of the treatment strategies for managing diabetes since these enzyme controls the catabolism of starch into glucose [43]. In the present study, the observed appreciable α-amylase inhibitory activity suggests that the plant may exhibit an antidiabetic effect by potentially reducing postprandial glucose absorption. This conclusion is supported by the lack of a significant difference observed between the positive control, acarbose (26.72 ± 3.59), and the experimental plant, *R. abyssinica* (21.37 ± 4.25 μg/ml μg/ml). This effect might be due to the presence of chemical constituents, terpenes, flavonoids [44]. These secondary metabolites are a major group of polyphenolic compounds that have been reported to possess inhibitory activity against α-amylase [45].

Given the promising effect of the extracts in reducing the *in vitro* alpha-amylase activity, further study was conducted to evaluate its potential *in vivo* hypoglycemic in normal mice and antihyperglycemic effect in glucose loaded and STZ induced diabetic mice. Prior to initiating the main *in vivo* studies, an initial acute toxicity study was conducted. The results indicated that administration of 70 % ethanolic fruit extracts of *R. abyssinica* did not produce any observable signs of behavioral changes or toxicity. Consequently, the LD50 of the experimental plant was determined to be greater than 2000 mg/kg. In the normoglycemic study, except with the highest dose of extract, RAFE400, at 3rd and 4th hr (P <0.05), the other doses of 70 % ethanol fruit extract of *R. abyssinica*, did not produced apparent hypoglycemic effect. On the other hand, the standard drug (GLI5) reduced BGL consistently at all-time points. These might suggest that the plant extract at its highest dose could have similar activity to glibbenclamide. In assessing antihyperglycemic activity through an OGTT, mice were initially fasted for 14 h before glucose administration. This approach is physiological induction of DM, as it temporarily elevates BGL in laboratory animals without damaging the pancreas [46]. It is commonly used to monitor how blood glucose homeostasis is maintained following glucose overload. This test complements glycemia monitoring in diabetes care and may be essential for detecting subtle changes during the progression of insulin resistance [47]. In this model, the 70 % hydroethanolic crude extract of *R.abyssinica* was found to have glucose lowering effect after oral administrations of 2.5 g/kg glucose solution. The noticeable reduction in BGL from the extract began within 30 min after glucose loading at the highest dose, and by 60 min across all dose levels. This effect persisted and was still observable at the 120-min. The reduction of BGL following glucose challenge may be due to the presence of hypoglycemic bioactive molecules [48].

In chemical induced DM model, STZ is employed. This chemical selectively destroys the pancreatic insulin-secreting β-cells, leaving less active cells and resulting in DM [49,50]. Following its uptake into the beta cells, STZ degrades into its glucose and methylnitrosourea moiety. Owing to its alkylating properties, methylnitrosourea moiety modifies biological macromolecules, fragments DNA and destroys the beta cells, causing a state of insulin-dependent diabetes [51]. In the present study, STZ-induced diabetic mice showed significant increase in glucose levels when compared to normal mice. The increased levels of plasma glucose were decreased upon treatment with hydroalcoholic extracts of *R.abyssinica*. In the Single-dose study, the two higher doses of the extract, RAF200 and RAF400, brought the BGL near to the normal range starting at 3rd and 4th hr (p˂0.001). The antihyperglycemic activity effect witnessed from these two dose levels of the plant extract is comparable with regard to the positive control group, GLI5. In the antihyperglycemic activity of repeated daily doses of the extract, the maximum percent fall of FBG was found with RAFE400 (72.95 %) followed by RAFE200 (62.53 %) at the end of third week. The result was comparable with the standard drug GLI5 that brought the glucose level to 85 mg/dl (77.66 %) on the same day. Except the lowest dose of plant extract, which reduced FBG only at the end of treatment period, RAFE200 and RAFE400 tend to show a significant reduction at second week compared to the negative control groups. In terms of the time required to bring FBG back to normal, it took only two weeks for the highest dose of extract while RAFE200 normalizes BGL at the third week. The observation revealed that the extract's effectiveness followed a dose-dependent pattern. Similarly, glibenclamide experienced in turning FBG level below 200 mg/dl starting from second week of treatment periods. The standard drug used in this model (glibenclamide) stimulates insulin release by binding to a specific site on the β cell of adenosine triphosphate sensitive K+ (KATP) channel complex and inhibiting its activity. KATP channel inhibition causes to membrane depolarization, activate voltage-gated Ca2+ channels, a rise in cytosolic (Ca2+) and release of endogenous insulin [52]. Furthermore, a single high dose of STZ might not cause complete destruction of β-cells/or few cells remained to have the capability to regenerate and secrete insulin [53]. Scholars also showed an increase in β-cell density per area of islet in diabetic rats, suggesting initiation of a compensatory response to STZ damage by surviving pancreatic β-cell [54]. These suggested that the antidiabetic effect of 70 % ethanolic fruit extracts of *R. abyssinica* might be due to potentiation of insulin effect probably by increasing secretion of insulin from the remaining β–cells or by increasing the peripheral glucose uptake.

Apart from hyperglycemia, STZ causes ulcers, hepatotoxicity, nephrotoxicity, and severe weight loss [55]. Loss of body weight after STZ administration is due to dehydration and catabolism of fats and protein [56]. In the present study, the experimental animals in the negative control group exhibited a significant weight reduction starting from week 1 compared to the normal control group. Conversely, administrations of GLI5, RAFE200 and RAFE400 for three consecutive weeks rescued body weight loss in contrast with negative control group. This could be attributed to the improvement of glycemic control and structural protein synthesis [27].

The *in vivo* antihyperglycemic effects of the plant extract are consistent with previous studies, such as Kifle et al. (2020). [57], which demonstrated the antidiabetic effects of *Hagenia abyssinica*, and Bahrami et al. (2021), which showed that other herbal products in the Rosaceae family, including *Malus domestica* (apple), *Cydonia oblonga* (quince), and *Prunus persica* (nectarine), produced antihyperglycemic effects in glucose-loaded and STZ-induced diabetic models [58].

The blood glucose lowering effects of the experimental plant, *R. abyssinica* is attributed to its secondary metabolites [59]. For example, tannin improves the function of pancreatic beta-cells and increases insulin release and the antidiabetic activity of alkaloids is through the inhibition of enzymes such a α-amylase, α-glucosidase and dipeptidyl peptidase-IV, as well as inhibition of advanced glycation end products [60]. On the other hand, antidiabetic activities of saponins are associated with regulating BGL and prevent diabetic complications due to their antioxidant effect [61]. In the phytochemicals screening of secondary metabolites, *R. abyssinica* contains alkaloids, phenols, tannins, glycosides, steroids and terpenoids. Furthermore, quantitative estimations of secondary metabolites unveiled the current experimenat plant contains alkaloids (83.37 mg ATP/g), phenols (892 mg GAE/g) including flavonoids (286.58 mg QC/g).

## 11 Conclusion

The result of this study revealed that ethanolic fruit extract of *R.abysssinica* has significant anti-diabetic effect. The results obtained from the *in vitro* studies suggested that the extract reduced plasma glucose level through decreasing post-prandial hyperglycemia via α-amylase inhibition activity. The *in vivo* study, on the other hand, revealed that oral administrations of the extract of *R.abyssinica* has a beneficial effect in reducing blood glucose level in both oral glucose loaded and streptozotocin-induced diabetic mice.

## 12 Limitations of the study

This study confirmed the antidiabetic activity of the 70 % fruit extract of *R. abyssinica*, demonstrating its ability to reduce postprandial hyperglycemia through α-amylase inhibition *in vitro* and lower BGL *in vivo* in both glucose-loaded and STZ-induced diabetic mice with minimal risk of hypoglycemia. However, the study's limitations include the lack of identification of specific bioactive compounds responsible for the antidiabetic action, absence of long-term efficacy and safety data, and missing histopathological analysis of major organs. Further study is recommended to explore the exact mechanism of action, evaluate the plant's antioxidant properties, and assess the effects of repeated dosing and potential impacts on major organs.

## Ethical approval

The study protocol was approved by the Institutional Review Board of the School of Pharmacy, College of Health Sciences, Addis Ababa University (reference no. ERB/SOP/466/14/2022). Furthermore, all experiment and procedures on animal were carried out in compliance with the globally recognized guidelines for the use, care and welfare of laboratory animals.

## Funding

The study was done with funding obtained from Addis Ababa University for Msc dissertation research. The role of the funder on the research was providing animals used in the experiment and financial support for purchasing materials and chemicals.

## Consent for publication

Not applicable.

## Data availability

The datasets used in this study are available from the corresponding author upon request.

## Authors contribution

All authors made a significant contribution to the work reported, whether that is in the conception, study design, execution, acquisition of data, analysis and interpretation, or in all these areas; took part in drafting, revising or critically reviewing the article; gave final approval of the version to be published; have agreed on the journal to which the article has been submitted; and agree to be accountable for all aspects of the work.

## CRediT authorship contribution statement

**Mohammed Ahmed Abdul:** Writing – review & editing, Writing – original draft, Visualization, Validation, Supervision, Software, Resources, Project administration, Methodology, Investigation, Funding acquisition, Formal analysis, Data curation, Conceptualization. **Akeberegn Gorems Ayele:** Writing – review & editing, Writing – original draft, Visualization, Validation, Supervision, Software, Resources, Project administration, Methodology, Investigation, Funding acquisition, Formal analysis, Data curation, Conceptualization. **Frehiwot Teka:** Writing – review & editing, Writing – original draft, Visualization, Validation, Supervision, Software, Resources, Project administration, Methodology, Investigation, Funding acquisition, Formal analysis, Data curation, Conceptualization. **Worku Gemchu:** Writing – review & editing, Writing – original draft, Visualization, Validation, Supervision, Software, Resources, Project administration, Methodology, Investigation, Funding acquisition, Formal analysis, Data curation, Conceptualization. **Workineh Shibeshi:** Writing – review & editing, Writing – original draft, Visualization, Validation, Supervision, Software, Resources, Project administration, Methodology, Investigation, Funding acquisition, Formal analysis, Data curation, Conceptualization.

## Abbreviations

BGL, blood glucose level; DM, Diabetes mellitus; DNS, Dinitrosalicylic acid; FBG, Fasting blood glucose; FC, Folin-Ciocalteu Fasting blood glucose; GAE, Gallic acid equivalent weight; KATP, Adenosine triphosphate sensitive K+; OGTT, Oral glucose tolerance; STZ, Streptozotocin.
